# Cell-free nucleic acids as noninvasive biomarkers for colorectal cancer detection

**DOI:** 10.3389/fgene.2014.00182

**Published:** 2014-08-27

**Authors:** Hicham Mansour

**Affiliations:** Biosciences Core Laboratories, Research Department, King Abdullah University of Science and TechnologyThuwal, Kingdom of Saudi Arabia

**Keywords:** colorectal cancer, cell-free nucleic acids, CFNA, biomarkers, noninvasive tests, miRNAs, DNA mutation, DNA methylation

## Abstract

Cell-free nucleic acids (CFNA) have been reported by several authors in blood, stool, and urine of patients with colorectal cancer (CRC). These genetic biomarkers can be an indication of neoplastic colorectal epithelial cells, and can thus potentially be used as noninvasive tests for the detection of the disease in CRC patients and monitor their staging, without the need to use heavier and invasive tools. In a number of test-trials, these genetic tests have shown the advantage of non-invasiveness, making them well accepted by most of the patients, without major side effects. They have also shown a promising sensitivity and specificity in the detection of malignant and premalignant neoplasms. Moreover, costs for performing such tests are very low. Several studies reported and confirmed the proof of the principle for these genetic tests for screening, diagnosis, and prognosis; the main challenge of translating this approach from research to clinical laboratory is the validation from large and long-term randomized trials to prove sustainable high sensitivity and specificity. In this paper, we present a review on the noninvasive genetics biomarkers for CRC detection described in the literature and the challenges that can be encountered for validation processes.

## Introduction

Colorectal cancer (CRC) is the most common cancer worldwide, and it is the second cause of cancer death just behind lung cancer (Ferlay et al., 2010). Recent data collected by Globocan, show an estimated 1.3 million new CRC cases during 2012, 693,881 resulting in death (http://globocan.iarc.fr/Pages/online.aspx). The CRC incidence and mortality are actually increasing despite recent advances in surgery, radiotherapy and chemotherapy (Boyle and Langman, 2000). Tumor progression of CRC is governed by either genetic or epigenetic changes intrinsic to neoplastic colorectal epithelial cells. CRC can be caused by changes in different molecular pathogenic pathways, such as chromosomal instability, CpG island methylator phenotype, and microsatellite instability. The majority of the cases are sporadic (not inherited). Sporadic changes may be acquired from diverse factors such as environmental exposures, diet, hormones, and normal aging. There are also cases of inherited CRC, the most known hereditary CRC being Familial adenomatous polyposis (FAP), Hereditary nonpolyposis colorectal cancer (HNPCC), Autosomal recessive adenomatous polyposis, and Oligodontia-colorectal cancer syndrome (Haggar and Boushey, 2009; Schweiger et al., 2013). Mutations in a single gene such as APC, MSH2, MLH1, PMS1, PMS2, MSH6, TGFBR2, MLH3, MUTYH, and AXIN2 result in a marked predisposition to mentioned hereditary CRC (Schweiger et al., 2013).

In most cases, the natural history of CRC follows a progression from benign polyps to advanced CRC, this process often spanning several years. Tumor detection at early disease stages together with monitoring of disease progression toward malignancy, have shown a reduction in mortality rates (Scholefield et al., 2012; Shaukat et al., 2013). Thereby, tumor control leads to better care of affected patients. Colonoscopy is currently the standard approach to tumor diagnosis, with a relatively high cost and recognized invasiveness with the potential for serious complications. Practical limitations restrict the use of colonoscopy for screening. An alternative method, Fecal Occult Blood Testing (FOBT) has been widely used to detect blood in fecal samples. The FOBT employs the pseudoperoxidase activity of hemoglobin's heme moiety in blood containing samples. More recently, the use of an immunological reaction directed to detect hemoglobin via heme-specific antibody has served as an alternative to the enzyme detection method. Unfortunately, several trials have shown the occurrence of a high rate of false-negatives and positives for these tests (Imperiale et al., 2004; Ebert et al., 2006; Bin Raies et al., 2013; Mansour et al., 2013; Roperch et al., 2013), Therefore, the need to develop an effective noninvasive assay to screen patients for CRC at early stages remains urgent.

## CFNA markers in body fluids

### Outlook

Since the first discovery in 1948 by Mandel of the occurrence of CFNA in blood (Mandel and Metais, 1948), researchers have found nucleic acids in body fluids that track and discriminate affected patients from healthy subjects. In the cancer field, researchers have also found traces of shed tumoral-nucleic acids in different biological effluents such as stool, blood, and urine. The recognition of the advantages and the proof-of-concept of using CFNAs for noninvasive cancer detection naturally followed their discovery (Imperiale et al., 2004; Muller et al., 2004; Ebert et al., 2006; Mansour and Sobhani, 2009; Roperch et al., 2013).

### Selection of literature

We performed a literature search to capture identified tumor-related CFNAs reported to distinguish CRC patients from healthy subjects. Thereby, a search from the NCBI Entrez search line selected to the PubMed index was performed with the following string: (plasma OR serum OR stool OR urine OR blood OR body fluids) AND (colorectal cancer OR colorectal neoplasms OR colorectal neoplasm OR colon cancer OR rectal cancer) AND (mutation or methylation or microRNA OR transcript OR mRNA OR miR OR RNA). The search yielded 3674 entries. The addition of the term “noninvasive” to the search string reduced the number of resulting titles to 109. The output data identified by these later inclusion criteria are reviewed and summarized in Table 1.

**Table 1:** **Summary of genetic markers found in body fluids from CRC patients**.

**Anomalies**	**Genetic marker**	**Body fluid**	**Sensitivity**	**Specificity**	**Method**	**References**
Mutation (codons 12 or 13)	K-ras	Stool	66.70%	100%	Hybridization assays and Southern-blot	Sidransky et al., 1992
Expression	PKC isoforms	Feces	ND	ND	Immunoblotting and mRNA directed PCR	Davidson et al., 1994
Mutation (codon 12 or 13)	K-ras	Stool	18.10%	ND	MASA-PCR method and gel blotting	Hasegawa et al., 1995
Mutation (Asp^13^, Val^12^, Asp^12^)	K-ras	Stool	29.90%	95.7%	PCR and Oligomer-specific hybridization	Villa et al., 1996
Mutation (codon 12 or 13)	K-ras	Stool	40%	100%	PCR and Restriction enzyme analysis	Ratto et al., 1996
Expression (variant 6 and 10)	Cd44	Stool	60–68%	ND	RT-PCR followed by Southern-blot	Yamao et al., 1998
Mutation (exon 4, 5, 6, 7 and 8)	P53	Blood	0% dukes stage A, 11% B, 18% C,	ND	Antibody Ber EP4 selection, RT-PCR and sequencing	Khan et al., 2000
Mutation (exons 5–8)	P53	Stool	0% dukes' stage A, 5% B, 5% C, and 33% D	100%	PCR followed by denaturing gradient gel electrophoresis and sequencing	Rengucci et al., 2001
Mutation (exons 1–2)	Kras		0% dukes' stage A, 10% B, 20% C, and 0% D	100%		
Microsatellites instability	D2S123, D5S346, D17S250, BAT25, BAT26		0 % dukes' stage A, 5% B, 5% C, and 33% D	100%		
9 mutations	TP53	Stool	71% (dukes' A 100%, B 82%, C 67%, and D 58%)	ND	Mismatch-ligation assay, modified solid-phase mini-sequencing method and Digital PCR-based method	Dong et al., 2001
Deletion	Bat26					
Mutations (codons 12–13)	Kras					
Mutation (GAT/TGT/GTT/AGT/GAC)	Kras	Stool	100% (6/6 found in tissue)	100%	Enrichment by biotinylated primers and streptavidin beads followed by Single-Strand Conformational Polymorphism	Doolittle et al., 2001
27 Mutations (codons 1210 and 1581)	APC	Stool	57% neoplasia (17/28 dukes'B2 and 9/18 adenomas)	100%	Digital protein truncation (*in vitro* transcription and translation of amplified-DNA)	Traverso et al., 2002
Microsatellites instability and lost of heterozygosity	P53	Stool	P53: sporadic cancer: 86.7% (26/30) and HNPCC 36.3% (4/11),	86.70%	PCR and fragment analysis	Koshiji et al., 2002
	APC		APC: sporadic cancer: 76.6% (23/30) and HNPCC 54.5% (6/11),			
	D9S162		D9S162: sporadic cancer: 73.3% (22/30) and HNPCC 54.5% (6/11). P53 and APC: 96.7% (sporadic cancer) P53, APC, and D9S162: 100% (sporadic cancer)			
	D9S171		Sporadic cancer: 36.6% (11/30) and HNPCC 36.3% (4/11)	ND		
	hMLH1		sporadic: 70% (21/30) and HNPCC 100% (11/11)	ND		
	IFNA		sporadic: 66.6% (20/30) and HNPCC 72.7% (8/11)	ND		
	DCC		sporadic: 53.3% (16/30) and HNPCC 81.8% (9/11)	ND		
Mutation in the first or second position of codon 12	Kras	Sera	31% (5/16) of carcinoma and 50% (2/4) ulcerative pancolitis, 0% adenomas, 0% Crohn disease	100%	RFLP-PCR	Borchers et al., 2002
Microsatellite instability (Deletion)	BAT-26	Stool	83% of successfully amplified samples. P53 (42%), Apc (37%), K-ras (28%), and BAT-26 (24%)	ND	PCR	Berger et al., 2003
Mutation (in 19 loci)	P53, K-ras, Apc					
Mutation in exons 5-8	P53	Stool		ND	ND	Calistri et al., 2003
Microsatellite instability (5 loci)	ND	6%				
Mutation in exons 1-2	Kras		11%			
Mutation (4 fragments in exon 15)	APC		2%			
Expression of telomersae	hTERT	Plasma	98%	64%	qRT-PCR	Lledo et al., 2004
Methylation	SFRP2	Stool			QPCR	Muller et al., 2004
CpG island Methylation	ESR1	Stool	ND	ND	Methylation-specific PCR and Cobra assay	Belshaw et al., 2004
	MGMT		ND	ND		
	HPP1		ND	ND		
	p16(INK4a)		ND	ND		
	APC		ND	ND		
	MLH1		ND	ND		
Muattion (21 mutations)	Kras, APC and P53	Stool	51.6 % (16/31) invasive cancers, 40.8% (29/71) invasive cancers plus adenomas with high-grade dysplasia and 18.2% (76/418) advanced neoplasia	94%	Oligonucleotide-based hybrid captures in DNA extraction followed by Specific PCR and capillary sequencing or Real Time PCR	Imperiale et al., 2004
Microsatellite instability	BAT-26					
DNA degradation marker	long DNA					
Methylation	APC	Serum	57 % (28/49) with at least one marker	95 %	Quantitative methylation-specific PCR (MethyLight PCR)	Leung et al., 2005
	hMLH1					
	HLTF					
Mutation (22 mutations)	Kras, APC, P53, bat-26	Stool	72%	ND	DNA analyzed gel-based capture	Itzkowitz et al., 2007
DNA integrity assay (DIA)	Long DNA			ND		
Methylation	Vimentin		72.50%	86.90%		
Mutation in codon 12	Kras	Stool	41% (12/29)	95%	Nested RT-PCR and amplified restriction fragment length polymorphism analysis	Chien et al., 2007
Mutation in codon 12	Kras	Stool	54% (14/26)	ND	Restriction endonuclease-mediated selective (REMS)-PCR	Mixich et al., 2007
Methylation	SFRP2	Stool	94.2% cancer, 52.4% adenoma, 28.5% *H. polyps* and *U. colitis*	95.80%	MSP	Huang et al., 2007.
DNA integrity	Long DNA	Stool	64%	95.00%	PCR, denaturating polyacrylamide gel, and MSP	Abbaszadegan et al., 2007
Methylation	P16		20%	100.00%		
Microsatellite instability	Bat-26		0%	100.00%		
Methylation	SFRP2	Stool	89%	86.00%	Methylation-specific polymerase chain reaction	Zhang et al., 2007
Methylation	SFRP2	Stool	87.0% (60/69) CRC, 61.8% (21/34) adenoma and 42.3% *H. polyp* (11/26)	93.00%	MethyLight PCR	Wang and Tang, 2008
Methylation	TFPI2	Stool	76–89%	79–93%	Quantitative methylation-specific PCR	Glockner et al., 2009
Methylation	GATA4	Stool	71% (in the training set) and 51% in the (validation set)	84% in the 1st set and 93% in the 2^d^ set	Quantitative MSP	Hellebrekers et al., 2009
Methylation	NDRG4	Stool	61% (training set) and 53% (test set)	93% (training set) and 100% (test set)	Quantitative MSP	Melotte et al., 2009
Methylation	MGMT	Stool	75.0% for CRC and 59.6% for adenoma	86.50%	MSP	Baek et al., 2009
	hMLH1					
	Vimentin					
Methylation	RASSF2	Stool	75.0% colorectal cancer and 44.4% advanced colorectal adenomas	89.40%	Single-step modification of DNA with sodium bisulfite and fluorescence polymerase chain reaction methodology	Nagasaka et al., 2009
	SFRP2					
Methylation	RARB2	Stool	In the initial set:75% of carcinomas, 60% of adenomas; in replication set: 62% of carcinomas et 40% of adenomas	100.00%	Methylation-specific melting curve analysis (MS-MCA)	Azuara et al., 2010
	p16INK4a					
	MGMT					
	APC					
Methylation	ALX4	Plasma	81%	90.00%	MethyLight PCR	He et al., 2010
	Sept9					
	TMEFF2					
Mutation	Kras	Stool	56.60%	93.30%	Chip-based temperature gradient capillary electrophoresis (TGCE)	Zhang et al., 2011a
Methylation	Mal	Stool	92.8% colorectal cancer, 70.8% in colon adenomas	96.20%	Methylation-specific PCR(MSP)	Kang et al., 2011
	CDKN2A			100.00%		
	MGMT			96.20%		
Methylation	TFPI2	Stool	86.70%	83.30%	Methylation-specific PCR (MSP)	Zhang et al., 2012
DNA integrity	Long DNA					
Methylation	Vimentin	Stool	86.7% CRC and 76.5% for adenoma	86.70%	Methylation-specific polymerase chain reaction (MSP)	Zhang et al., 2011b
	OSMR					
	TFPI2					
Mutation (3925 G > A, 4012 C > T, 4067 C > T, and 4099 C > T)	APC	Stool	50%	ND	Hydrogel bead-array	Deng et al., 2012
Mutation (814 G > A and 818 G > A)	TP53					
Mutation (35 G > T and 38 G > A)	Kras					
SERS spectra	RNA	Serum	89.10%	95.60%	Surface-enhanced Raman scattering (SERS), platform	Chen et al., 2012
Chromosomal alterations	Whole genome	Plasma	100%	100%	NGS	Leary et al., 2012
Methylation	Spastic paraplegia-20	Stool	80.20%	100%	Methylation specific PCR	Zhang et al., 2013a
Methylation	AGTR1	Stool	78.00%	ND	Methylation array and pyrosequencing	Carmona et al., 2013
	WNT2					
	SLIT2					
Methylation	FBN1	Stool	72.00%	93%	Methylation-specific PCR	Guo et al., 2013
Mutation	APC	Stool	ND	ND	Wild-type blocking PCR and high-resolution melting (WT-HRM)	Gerecke et al., 2013
Mutation and Methylation	KRAS mutations, aberrant NDRG4 and BMP3 methylation, and β-actin	Stool	92.3% colorectal cancer, 42.4% advanced precancerous lesions, 69.2% polyps with high-grade dysplasia, 42.4% serrated sessile polyps measuring 1 cm or more	86%	Quantitative Molecular Assays	Imperiale et al., 2014
MicroRNA expression	miR-532-3p	Plasma	Polyps discrimination from controls with high accuracy	ND	Microfluidic array technology	Kanaan et al., 2013
	miR-331					
	miR-195					
	miR-17					
	miR-142-3p					
	miR-15b					
	miR-532					
	miR-652					
	miR-29a	Plasma	83%	85%	RT-PCR	Huang et al., 2010
	miR-92a	Plasma				
	miR-21	Stool	ND	ND	TaqMan quantitative reverse transcription-PCR	Link et al., 2010
	miR-106a					
	miR-221	Plasma	ND	ND	Quantitative Reverse Transcription-Polymerase Chain Reaction	Pu et al., 2010
	miR-144	Feces	74%	87%	RT-qPCR	Kalimutho et al., 2011a
	miR-21	Plasma	90%	90%	Microfluidic Array Technology	Kanaan et al., 2012
	miR-18a, miR-19a, miR-19b, miR-15b, miR-29a, and miR-335	Plasma	ND		Quantitative reverse-transcription PCR	Giraldez et al., 2013
	miR-532-3p, miR-331, miR-195, miR-17, miR-142-3p, miR-15b, miR-532, and miR-652	Plasma	[area under curve (AUC) = 0.868 (95% confidence interval [CI]: 0.76–0.98)]		Microfluidic Array Technology	Kanaan et al., 2013
	miR-21, let-7g, miR-31, miR-92a, miR-181b, and miR-203	Serum	Areas under ROC curve were 0.900 and 0.923 for the two sets of samples		Quantitative Reverse Transcription Polymerase Chain Reactions	Wang et al., 2014
	miR-135b	Plasma	78% for CRC, 73% for advanced adenoma, and 65% for any adenoma	68%	microRNA expression array	Wu et al., 2014b
	miR-18a	Plasma	ND	ND	Microfluidic Array Technology	Komatsu et al., 2014
	miR-92	Plasma	89%	70%	Real-Time PCR	Ng et al., 2009
	miR-141	Plasma	ND	ND	Quantitative Reverse Transcription-Polymerase Chain Reaction	Cheng et al., 2011
	miR-601, miR-760	Plasma	83%	69%	qRT-PCR	Wang et al., 2012
	miR-18a, miR-20a, miR-21, miR-29a, miR-92a, miR-106b, miR-133a, miR-143, miR-145	Plasma	ND	ND	TaqMan MicroRNA Array	Luo et al., 2013
	miR-378	Plasma	ND	ND	Quantitative Real Time PCR	Zanutto et al., 2014
	miR-200c	Plasma	84.60%	75.60%	ND	Zhang et al., 2013b
	miR-18a					
	RNU2-1f (Circulating U2 small nuclear RNA)	Plasma	98%	91%	qRT-PCR	Baraniskin et al., 2013
	let-7a, miR-1229, miR-1246, miR-150, miR-21, miR-223, and miR-23a	Serum	ND	ND	Microarray analyses and Real Time PCR	Ogata-Kawata et al., 2014
	miR19a	Plasma	80.95% for TNM I/II, 76.19% for TNM III/IV	79.25–77.36%	Genome-wide miRNA expression profiling assay and qRT-PCR	Giraldez et al., 2013
	miR19b					
	miR15b					
	miR-21	Serum	90%	90%	Microfluidic array technology	Kanaan et al., 2012
	miR-29a	Plasma	83% for CRC and 73% for advanced adenomas	84.7%-79.7%	Real Time PCR	Huang et al., 2010
	miR-92a					
	miR-21	Stool	Higher expression	ND	Taqman-RT-PCR	Link et al., 2010
	miR-106a					
	miR-144	Stool	74%	87%	RT-pre-amplification-qPCR	Kalimutho et al., 2011a
	miR-221	Plasma	86%	41%	Quantitative Reverse Transcription-Polymerase Chain Reaction without RNA extraction	Pu et al., 2010
MicroRNA Methylation	miR-34a	Stool	77%	94%	Methylation-Specific PCR	Wu et al., 2014a
	miR-34b/c		95%	100%		
	miR-34b/c	Stool	75%	ND	Methylation-Specific PCR	Kalimutho et al., 2011b
	miR-34b/c	Stool	ND	ND	ND	Cho, 2011
	miR-148a					

### Literature search results

This section is organized as follows: We first describe the different available molecular approaches for the detection of anomalies in CFNA in body fluids, we then review the inclusion criteria for the patient cohorts and we finally describe the known CFNA biomarkers.

#### Molecular approaches

Several molecular approaches are used to detect the CFNA in the body fluids of CRC patients and to quantify the molecular anomalies that could reveal neoplastic traces. The quantitative polymerase chain reaction (QPCR or RT-PCR) is widely used. Other tools are also reported such as DNA Beaming Technology, Methylation Array, DNA array, Surface-Enhanced Raman Scattering (SERS), and Restriction Fragment Length Polymorphism (RFLP). While most of these methods capture and enrich the tumoral nucleic acids to bring their levels to the detection and quantification thresholds, the sensitivity of these instruments, and techniques vary considerably with a resulting influence on the performance of the test to distinguish the cancer from the normal. This variability was observed even when evaluating the same marker. One new molecular approach for evaluation the whole CFNA that can be found in the body fluids is the use of next generation sequencing (NGS), as in Leary et al. (2012). They scanned the whole genome of plasmatic DNA to detect which composites of chromosomal structure variations present in CRC tumorigenesis could discriminate between CRC patients and healthy subjects. Promising results were found discriminating CRC patients at advanced stage from healthy controls, but these results would benefit from a more in-depth study on a large cohort and at earlier disease stages. This study did emphasize the value of using NGS to CRC detection. NGS applied to CRC detection could provide high sensitivity through high sequencing coverage and selectivity through complete analysis of high quality data.

#### Patients

In term of patients' inclusion and tumor criteria, the majority of these studies used average-risk population. Different tumor sizes were considered: small adenomas, adenomas having more than 1 cm in size and carcinomas at different stages (I, II, III, or IV). The cancer locations were mainly ascending colon, transverse colon, descending colon, sigmoid colon, and rectum. Three clinical trials differed strongly from all the others. The first one was a study from Manenti et al. (Villa et al., 1996), focusing on Kras gene mutations in the stool of 230 consecutive patients. The consecutive patient selection strategy gives unbiased sensitivity and specificity regarding the test used and clearly aims to avoid bias possibly introduced by patient selection. The two others outstanding studies were performed by Imperiale et al. They evaluated 21 mutations in APC, Kras, and P53 genes on asymptomatic subjects (*n* = 4404) tested already for FOBT (Imperiale et al., 2004), and they recently evaluated KRAS mutations, aberrant NDRG4 and BMP3 methylation, and β-actin on very large number of individuals (*n* = 9989) tested for FIT (Imperiale et al., 2014). Inclusion of a very large number of asymptomatic subjects shows the potential of sensitivity gain for molecular testing (Imperiale et al., 2004, 2014). With these inclusion criteria, the authors simulated a real situation of screening and applied powerful statistical tools to evaluate the true performance of the test used. Globally, the authors claim a higher sensitivity for the multitarget stool DNA testing over the two tests FOBT and the FIT, but had more false positives results (Imperiale et al., 2004, 2014). Other authors have identified, albeit in small cohorts, several others combinations of useful biomarkers. Studies directed at larger cohorts are awaited to provide confidence on the performance of the composite panels that were chosen for these studies.

#### Biomarkers

The first results to provide a conceptual framework and a practical basis for a new molecular approach to detect the presence of genetic anomalies in CFNAs isolated from CRC patient stool samples were published in 1992 by Bert Vogelstein et al. (Sidransky et al., 1992). In this work, the authors were able in a relatively easy manner, to detect *K-ras* gene mutations in patients with CRC. They successfully detected Kras mutation in 89% of the patients. Since this first published results targeting Kras, other anomalies were measured directly from CFNAs including deletions, microsatellite instability, loss of heterozygosity, copy number variation, chromosomal rearrangements, DNA, and microRNA methylation changes and mis-expression of mRNAs and microRNAs. Many genes have been shown affected in CRC, are involved in various critical signaling molecular pathways, but their diagnostic usefulness and specificity vary considerably. Here follows a description of the most common CFNA biomarkers, subdivided by subclasses, namely mutation, methylation, and microRNA.

## Mutation biomarkers

### Kras

Kras, a Kirsten ras oncogene, encodes a protein that is a member of the small GTPase superfamily. The protein is involved in many vital signaling pathways, including proliferation, differentiation, and senescence. A single amino acid substitution is responsible for an activating mutation. The transforming protein that results is implicated in various malignancies, including lung adenocarcinoma, mucinous adenoma, ductal carcinoma of the pancreas, and colorectal carcinoma (Kranenburg, 2005).

Bert Vogelstein et al. were able to detect Kras gene mutations in patients with CRC, through hybridization and southern-blot assays of the isolated CFNAs. Despite a small cohort (*n* = 9), successful detection of a Kras mutation in eight of nine patients was independent of tumor type being detected in both benign and malignant neoplasms. The use of Kras mutation for detection did not depend on the tumor localization as either distal or proximal colonic tumors were detected (Sidransky et al., 1992).

This study stimulated further research to assess Kras mutation in stool. In several research studies using small cohorts (<100), it was an overall concordance between tissue and stool for Kras genotype and it was possible to detect mutation even in 1000-fold excess of wild-type Kras (Mixich et al., 2007). The overall results showed that Kras mutations has 34–87.5% of sensitivity for detecting CRC patients and the specificity was very high, reaching in some studies 100% (Dong et al., 2001; Doolittle et al., 2001; Rengucci et al., 2001; Calistri et al., 2003; Chien et al., 2007; Mixich et al., 2007; Zhang et al., 2011a).

In large asymptomatic cohort, Kras mutations were found in the stool of 16.1% (5/31) of adenocarcinoma patients, 4.5% (18/403) of advanced adenoma, 2.9% of patients with minor polyps (49/648), and in 1.5% (22/1423) of subjects with negative findings on colonoscopy (Imperiale et al., 2004).

### APC

APC, an Adenomatous Polyposis Coli gene, encodes a tumor suppressor protein that acts as an antagonist of the Wnt signaling pathway, thus blocks epithelial cell proliferation. It is also involved in other processes including cell migration and adhesion, apoptosis, spindle assembly, and chromosome segregation. Defects in this gene cause FAP, an autosomal dominant pre-malignant disease that usually progresses to malignancy. Disease-associated mutations tend to be clustered in a small region designated the mutation cluster region (MCR) and result in a truncated protein product (Hanson and Miller, 2005).

APC mutations were analyzed in many studies. Except for the study of Calistri et al. who found a very low frequency of APC mutations with 2% detected in the stool samples (Calistri et al., 2003), many researchers found APC mutations occurred in more than 76% of the HNPCC patients and 54% of the sporadic CRC (Koshiji et al., 2002; Traverso et al., 2002). APC mutations were also detected in patients with minor polyps, but at low fraction (2.5%) (Imperiale et al., 2004). The specificity of APC mutations was found in more than 99% of the subjects with negative findings on colonoscopy (Koshiji et al., 2002; Traverso et al., 2002). The combination of P53 anomalies with APC mutations increased the sensitivity of cancer detection to 96.7% (Koshiji et al., 2002).

The APC gene can carry relatively highly aberrant methylation patterns on CpG islands. In DNA from tissue samples, Azuara et al. (2010) showed the prevalence of promoter hypermethylation in tumor biopsies for APC in 20% (50/250) of the samples tested. The analysis of 100 cases, paired normal mucosa yielded zero percentage of APC methylation.

In DNA from stool samples, the authors detected APC methylation in 37.5% (12/32) of patients with adenomas or carcinomas and no methylation was found in the 22 non-neoplastic subjects having either IBD or normal bowel (Azuara et al., 2010). However, when Belshaw et al. evaluated APC gene methylation in stool samples, they found no difference in methylation between the CRC patients (*n* = 21) and the healthy volunteers (*n* = 12) (Belshaw et al., 2004). The same conclusion resulted in the study of Leung et al. in the serum. They analyzed APC methylation in 49 CRC patients and 41 age-matched controls as determined with normal colonoscopy. There was no significant difference found in the concentration of methylated serum DNA between cancer patients and controls for APC gene (*p* = 0.21) (Leung et al., 2005).

### TP53

The TP53 gene encodes a tumor suppressor protein containing transcriptional activation, DNA binding, and oligomerization domains. TP53 responds to diverse cellular stresses to properly regulate expression of target genes, thereby inducing cell cycle arrest, apoptosis, senescence, DNA repair, or changes in metabolism. In addition, P53 appears to induce apoptosis through non-transcriptional cytoplasmic processes. Mutations in this gene are associated with a variety of human cancers, including hereditary cancers such as Li-Fraumeni syndrome (Toledo and Wahl, 2006).

TP53 mutations were found in DNA stool samples of 25.8% (8/31) of adenocarcinoma patients, 2.7% (11/403) of advanced adenoma, 0.8% (5/648) of patients with minor polyps, and in 1.1% (16/1423) of subjects with negative findings on colonoscopy (Imperiale et al., 2004). Higher detection rates were found in the study of Khan et al. (2000) and Dong et al. (2001). In the first study, they detected TP53 mutations in solid tumor samples of 46% (19/41) colorectal carcinoma patients and in peripheral blood samples of 42% (8/19) patients (Khan et al., 2000). In the study of Dong et al. the authors isolated DNA from paired stools and primary tumor samples from CRC patients. They detected TP53 mutations in the stools as well as in the tumors of 59% (30/51) of the CRC patients (Dong et al., 2001).

Poor performance of P53 mutations was seen in several studies. Calistri et al. (2003) analyzed TP53 exon 5–8 in the stool from 38 healthy individuals and paired stools and primary lesions from 56 CRC patients. While the detection sensitivity in the tissues was 34%, the sensitivity in the stools was less than 6% (Calistri et al., 2003). Calistri et al. detected P53 mutations in the tumors of 37% (17/46) of CRC patients and in the stool of only 6% (3/46) of the CRC cases (Rengucci et al., 2001).

The combination of P53 mutations with other markers increases the performance of the test to detect CRC patients. Koshiji et al. evaluated both the TP53 and APC mutations in the stool of 30 patients with sporadic CRC and 15 individuals without cancer. The combination of TP53 and APC detected the CRC patients with 97% of sensitivity and 100% of specificity (Koshiji et al., 2002).

### MMR genes

Mismatch repair genes play a key role in maintaining genomic stability, through participating in the mismatch repair pathway. The major eukaryotic MMR genes are MLH1, MLH3, MSH2, MSH3, PMS2, and MSH6 (from KEGG source record: ko03430). These genes contain microsatellites coding repeats (Fishel et al., 1993; Desai et al., 2000; Hansen et al., 2014). The deletion or insertion of one or two nucleotides in these repeats causes a frameshift mutation resulting in the production of a truncated and inactive protein that ultimately affects the MMR biological pathway (Iacopetta et al., 2010). Several other non-MMR genes were found to contain microsatellites repeats and exhibit repeats instability in CRC.

In many research studies, microsatellite instability was a rare event. Calistri et al. evaluated microsatellite instability using a set of five microsatellite markers (D2S123, D5S346, D17S250, BAT25, and BAT26) in 46 cases of CRC and 18 healthy individuals. In the healthy individuals, no genetic alterations in stool were detected. The diagnostic sensitivity of combining five microsatellites markers was 7% in stool-DNA (Rengucci et al., 2001). Imperiale et al. used Bat-26 as microsatellite-instability marker. They found Bat-26 deletions in the stool of 6.5% (2/31) of adenocarcinoma patients, 1.2% (5/403) of advanced adenoma, 0.6% (4/648) of patients with minor polyps, and in 1.1% (16/1423) of subjects with negative findings on colonoscopy (Imperiale et al., 2004). Albeit the low frequency of microsatellites instability, it is important to combine microsatellites with others markers such as P53 and Kras mutations, because microsatellites instability could be present in tumors lacking P53 and Kras mutations.

## Methylation biomarkers

DNA methylation is an epigenetic mechanism that occurs in gene promoter CpG sites, it is a well-known mechanism for transcriptional silencing. Methylation instability events are frequently observed in CRC, resulting in the inactivation of several tumor suppressor genes or the activation of some tumor-related genes. Thereby, methylation aberration quantification can be used in diagnostics and prognosis of CRC.

**Methylation in the stool**. Muller et al. were the first to detect the methylation anomalies in stool DNA of CRC patients using secreted frizzled-related protein gene 2 (SFRP2). SFRP2 is a member of the SFRP family that contains a cysteine-rich domain homologous to the putative Wnt-binding site of Frizzled proteins and acts as soluble modulators of Wnt signaling pathway. As shown by many studies, the methylation of this gene is a potential marker for the presence of CRC. In the study of Muller et al., the methylation of SFRP2 was assessed in two independent sets of patients (*n* = 23 and *n* = 26). SFRP2 methylation had a detection sensitivity of 90 and 77% in the training and test sets, respectively, and the specificity was found to be 77% (Muller et al., 2004). Similar results were found in other studies, methylated SFRP2 was found to occur in 87–94.2% of patients with CRC, 52.4–61.8% with adenomas and in 37.5–42.3% with hyperplasic polyps (Huang et al., 2007; Wang and Tang, 2008). In these studies only 5–7% revealed methylated DNA out the normal individuals tested (Huang et al., 2007; Wang and Tang, 2008). Another SFRP family member, secreted frizzled-related protein gene 1 (SFRP1) was detected methylated in the stool of CRC patients. SFRP1 methylation was found to be statistically highly significant between patients with colorectal neoplasia and the healthy group, a sensitivity of 89% and specificity of 86% for the detection of colorectal neoplasia was found for this gene (Zhang et al., 2007).

The methylation in the stool of several others markers was proposed as potential marker for CRC screening as single marker, TFPI2 with sensitivity of 68% and specificity of 100% in the cohort of 80 patients and 30 healthy controls (Zhang et al., 2012), P16 with specificity of 20% and a specificity of 100% (Abbaszadegan et al., 2007), Fibrillin-1 with sensitivity of 72% and specificity of 93% (Guo et al., 2013), TFPI2 with sensitivity of 76% to 89% and a specificity of 79–93% (Glockner et al., 2009), NDRG4 in the training set yielded a sensitivity of 61%, and a specificity of 93% and in an independent test set of patients the methylation of this gene yielded a sensitivity of 53% and a specificity of 100% (Melotte et al., 2009), the paraplegia-20 was found with a sensitivity and specificity of 80.2 and 100%, respectively (Zhang et al., 2013a).

To increase the sensitivity for calling true positives CRC patients, researchers tried several combinations of methylation markers. In 296 fecal samples, the combination of RASSF2 and SFRP2 methylation detected 75% of patients with CRC, and 44% of patients with advanced colorectal adenomas. Only 11% of the subjects without neoplastic or active diseases were positives for at least one marker (Nagasaka et al., 2009). RARB2, p16INK4a, and MGMT methylation were combined to APC methylation. The methylation of at least one marker was detected in the stools of 75% (9/12) and 61.5% (16/26) of patients with carcinomas and 60% (12/20) and 40% (8/20) of patients with adenomas in the initial set and second set, respectively. The specificity of the combined markers on healthy subjects was 100% (Azuara et al., 2010). The methylation of MGMT, hMLH1, and vimentin were detected, respectively, in 51.7, 30.0, and 38.3% of CRC, and in 36.5, 11, and 15.4% of colorectal adenomas, in combination the sensitivity were 75 and 59.6%. The specificity of the three combined markers was 86.5% (Baek et al., 2009). The combination of the vimentin methylation to the DNA test used by Imperiale et al. (2004), improved the sensitivity of CRC detection to 80%, however, the specificity was decreased (Itzkowitz et al., 2007). The diagnostic sensitivity by combining the following three markers AGTR1, WNT2, and SLT2 was 78% (Carmona et al., 2013). The methylation frequencies of MAL, CDKN2A, and MGMT were respectively, 78.3, 52.5, and 55.1% in CRC, 58.3, 41.7 and 37.5% in colon adenomas, 26.3, 15.8 and 10.5% in hyperplastic polyps, and 3.8, 0 and 3.8% in healthy controls. However, the sensitivity of the combination those three markers was 92.8% in CRC and 70.8% in adenomas, showing significantly higher than FOBT examination (Kang et al., 2011). The combination of vimentin, OMSR, and TFPI2 methylation on stool-DNA from 107 individuals detected 86.7% of CRC and 76.5%, the adenoma, the specificity was 86.7% (Zhang et al., 2011b).

**Methylation abnormalities were also reported in the serum** of CRC patients by many authors. Methylation abnormalities of APC, hMLH1, and HLTF were detected in the bloodstream of CRC patients. Overall, 57% of CRC patients had methylation in at least one marker and 95% of the normal subjects were not carrying the methylation in these genes (Leung et al., 2005). A developed assay, designed to simultaneously quantify the methylation of ALX4, SEPT9, and TMEFF2, was applied to 182 peripheral blood samples from CRC patients. Methylation of ALX4, SEPT9, and TMEFF2 as single marker occurred only in 48, 75, and 71% of the CRC patients, respectively. In combination, the sensitivity of the combined markers were improved to 81% and the specificity was 90% (He et al., 2010).

**Methylation anomalies were also detected in urine** from CRC patients. In a recent work, we investigated WIF1, ALX4, and vimentin methylation in either urine or serum samples of 247 patients (90 patients with neoplasia and 157 control subjects normal colonoscopy or having small adenomas less than 1 cm). Hypermethylation of Wif-1 had higher diagnostic sensitivity than Alx4 or vimentin. WIF1 methylation was observed in 26.7 and 32.6% in CRC cases (*p* < 0.001) and in 1.3% of the control patients (*p* < 0.001) in either urine or serum. Interestingly, the combination of serum and urine raised the neoplasia detection rate to 47.8% in CRC cases, compared to 2.5% in control patients (Mansour and Sobhani, 2009; Amiot et al., 2014).

## MicroRNA biomarkers

MicroRNAs are small non-coding RNAs that function at post-transcriptional level to regulate gene expression by binding the 3′-untranslated region (3′UTR) of the target transcript (Maqbool and Hussain, 2014). MicroRNA can be aberrantly expressed or methylated in tumors and can be found and quantified either in stool or plasma. These microRNAs signatures could be a good noninvasive tool for the CRC detection (Cho, 2011; Maqbool and Hussain, 2014).

**In the stool** the screening of feces for 648 microRNAs showed that 39% of all these microRNAs can be detected (Kalimutho et al., 2011a). High expression levels of miR-21, miR-106a, miR-221, miR-29, miR-92, miR-34a, miR-34b/c, miR148a, and miR-144 expressions were found in the stool from patients with CRCs compared with healthy individuals (Huang et al., 2010; Link et al., 2010; Cho, 2011; Kalimutho et al., 2011a,b; Wu et al., 2014a). Other microRNAs, such as miR-34a, miR-b/c, and miR-148a were also assessed in DNA stool from CRC patients but for their methylation (Cho, 2011; Kalimutho et al., 2011b; Wu et al., 2014a). Researchers demonstrated that either the expression or the methylation of some microRNAs in the stool can be used as potential markers for CRC detection.

**In the plasma**, many microRNAs analyzed show variable performance for discriminating CRC from normal. In 103 CRC patients and 37 healthy normal controls, the plasma level of miR-221 is shown as a potential biomarker for differentiating CRC patients from controls. At a specific cutoff value of expression, miR-221 has the sensitivity of 86% and specificity of 41% (Pu et al., 2010). In the study investigating 380 plasmatic microRNAs, miR-21 was found to differentiate CRC patients from controls with 90% of sensitivity and specificity (Kanaan et al., 2012). In the analysis of 196 plasma samples from 123 patients newly diagnosed with sporadic colorectal neoplasia, miR-18a, miR-19a, miR-19b, miR-15b, miR-29a, and miR-335 were significantly up-regulated in CRC patients (Giraldez et al., 2013). They differentiate patients with CRC from controls with area under curve (AUC) values ranging from 0.80 (95% confidence interval [CI], 0.71–0.89) to 0.70 (95% CI, 0.59–0.80). In this study the only marker for advanced adenomas (AAs) was miR-18a that was significantly up-regulated in AAs compared with controls; the AUC value was 0.64 (95% CI, 0.52–0.75) (Giraldez et al., 2013). The investigation of 380 plasmatic microRNAs, in a small cohort of patients, revealed a panel of eight microRNAs (miR-532-3p, miR-331, miR-195, miR-17, miR-142-3p, miR-15b, miR-532, and miR-652) that was found to distinguish polyps from controls with high accuracy [(AUC) = 0.868 (95% [CI]: 0.76–0.98)] (Kanaan et al., 2013). In several others studies, others microRNA, were found also in the plasma of CRC patients as promising markers, such as, let-7g, miR-31, miR-181b, miR-203, miR-135b, RNU2-1f, let-7a, miR-1229, miR-1246, miR-150, miR-223, and miR-23a (Ng et al., 2009; Huang et al., 2010; Pu et al., 2010; Cheng et al., 2011; Kalimutho et al., 2011a; Kanaan et al., 2012, 2013; Wang et al., 2012, 2014; Baraniskin et al., 2013; Giraldez et al., 2013; Luo et al., 2013; Zhang et al., 2013b; Komatsu et al., 2014; Ogata-Kawata et al., 2014; Wu et al., 2014b; Zanutto et al., 2014).

## CFNA markers translation from research to clinical use

The biomarkers described to this point promise to be an excellent way of detecting CRC, in the average-risk population. This population typically includes individuals who are more than 50 years old or are first-degree relatives of CRC-affected patients or patients with inflammatory bowel disease (IBD) possibly having previously undetectable mucosal alterations, a prior step to detectable premalignant neoplastic lesions. We summarize in the following the advantages of CFNA based CRC detection.

Genetic anomalies: (1) appear in a large fraction of sporadic non-hereditary tumors, (2) they appear in early stage during gut cells transition to tumors, (3) they are well defined and described, (4) a large number of cells carrying these anomalies are shed from the developing tumor and could be found in biological effluents, in particular stool, serum, and urine, (5) the genetic anomalies can be easily identified by simple, quick, and relatively inexpensive molecular approaches.

These noninvasive molecular approaches (1) show high sensitivity and specificity in tumor detection and staging, (2) a higher performance than the FOBT or FIT results, and (3) can be used both as prognostic factor to monitor the disease progression and therapy responsiveness, by expanding or refining the biomarker panel as our tumor-biology knowledge increases and evolves.

There are multiple screening approaches that were endorsed by the American Cancer Society (ACS) including stool or serum DNA testing, and several companies are currently developing tests based on these approaches. Nevertheless, studies published to date that focus on the biomarker validation in large and long-term randomized trials are rare and implementation in screening trials have not been seen. For such validation and implementation, some technical challenges could be encountered,

- The DNA present in biological effluents could be a limiting factor for the less sensitive molecular tools as the quantity of CFNA extracted varies; stool contains higher DNA quantity compared to serum and urine. From stool we can reproducibly get a few μ g of DNA per 100 mg of stool; however, human DNA represents merely 0.01% of the total stool DNA. The urine seems to carry the lowest quantity of DNA, typically only a few ng per ml of urine.
- The origin of circulating free DNA in body fluids is not yet well established. While the cell-free DNA found in CRC patients may come from highly proliferative neoplastic colonic cells, a normal cell (colonic cells or immune cells) origin has also been indicated. Normal cells have been also found to ubiquitously release DNA fragments (Stroun et al., 2000; van der Vaart and Pretorius, 2007). The presence of different non-tumor DNA sources may obscure the tumor DNA and make genetic biomarker detection difficult. Indeed, the quantity of circulating tumor nucleic acids found in body fluids of cancer patients may be very low and varies considerably.
  In plasma, Leary et al. showed that tumor DNA of cancer patients ranges from 1.4 to 47.9% of wild-type (Leary et al., 2012). Mouliere et al. showed that the quantity of mutant DNA in plasma can be found even higher than what were previously described showing a variation range from 0.13 to 68% in samples from mutation-positives CRC patients (Mouliere et al., 2013). This variation can be influenced by cancer staging, where advanced stages carry more cell free DNA than early stages, as was shown by Bert Vogelstein et al. who quantified the tumoral DNA extracted from plasma of CRC patients and reported that the means of mutated DNA found were 0.02, 0.04, 0.94, and 11.05% for adenomas, stage A, stage B, and stage D, respectively. The lowest quantity of mutant fragment that was seen is 0.001%. While the quantity of mutant fragment appears to correlate positively with the staging, the lowest quantity was not specific to any stage (Diehl et al., 2005).
  In stool, Sidransky et al. (1992) showed that the tumor mutated DNA represent a low rate, 4–8% the wild-type DNA of CRC patients. Thus, both in plasma and in stool, the high sensitivity of the molecular approach is needed to detect patients with presumably curable CRC.
- The quality of extracted CFNA can vary considerably since body fluids do not preserve cell-free nucleic acid integrity. Stabilization of the body fluids just after the collection is strongly recommended, especially for stool samples.
- Some body fluids can carry some inhibitors, such as food digestion products, bacterial contaminants, and nucleic acids released from others cells, that can reduce the CRC detection performance.
- The cell free nucleic acids found in blood, urine, and stool are in general fragmented DNA, less than 200 bp. Then, the evaluation of the size of extracted CFNA and targeting shorter regions should be considered to attain high performance of detection.
- High-Throughput Sequencing is a good tool that enables screening the whole CFNA. High diagnostic value could be achieved by using pair-end libraries and deep sequencing coverage. However, the sequencing cost is still too high to be implemented in routine as screening approach.

## Conclusion

Research studies have shown that many genetic markers were promising for CRC screening and diagnosis. A long term-clinical trial evaluating all these biomarkers in the same cohort and comparing stool, serum and urine is needed to select the best composite panel. In addition, the challenges encountered with nucleic acids extracted from body fluids need to be overcome to identify the standard protocol and the robust tool to implement these biomarkers as standard test for CRC screening, diagnosis, and prognosis.

### Conflict of interest statement

The author declares that the research was conducted in the absence of any commercial or financial relationships that could be construed as a potential conflict of interest.
